# Distinct effects of Fgf7 and Fgf10 on the terminal differentiation of murine bladder urothelium revealed using an organoid culture system

**DOI:** 10.1186/s12894-023-01338-y

**Published:** 2023-10-24

**Authors:** Kazuto Suda, Yuka Matsumoto, Takanori Ochi, Hiroyuki Koga, Nobutaka Hattori, Atsuyuki Yamataka, Tetsuya Nakamura

**Affiliations:** 1 Department of Pediatric General and Urogenital Surgery, Juntendo University School of Medicine, 2- 1-1 Hongo, Bunkyo-ku, Tokyo, 113-8421 Japan; 2 Department of Research and Development for Organoids, Juntendo University Graduate School of Medicine, 2-1-1 Hongo, Bunkyo-ku, Tokyo, 113-8421 Japan; 3 Department of Neurology, Juntendo University School of Medicine, Tokyo, Japan

**Keywords:** Bladder, Urothelium, Differentiation, Organoid, Fibroblast growth factor 7, Fibroblast growth factor 10, Peroxisome proliferator-activated receptor γ

## Abstract

**Background:**

Dysregulation of the terminal differentiation of bladder urothelium is associated with the pathogenesis of urinary tract disorders. Fibroblast growth factor (Fgf)7 and Fgf10 stimulate urothelial proliferation; however, their roles in cellular differentiation remain unclear. In this study, we used an organoid system to investigate the roles of these Fgfs in regulating bladder urothelium differentiation and identify their distribution patterns in the mouse bladder.

**Methods:**

Adult bladder epithelia (AdBE) isolated from adult mouse bladder tissues (AdBTs) were used to culture adult bladder organoids (AdBOs) in the presence of Fgf7 and Fgf10. The differentiation status of the cells in AdBTs, AdBEs, AdBOs, and neonatal bladder tissues (NeoBTs) was analyzed via quantitative real-time-PCR for the presence of undifferentiated cell markers (Krt5, Trp63, and Krt14) and differentiated cell markers (Krt20, Upk1a, Upk2, and Upk3a). Organoid cell proliferation was assessed by counting cell numbers using the trypan blue method. The effects of Fgf7 and Fgf10 on organoid differentiation were assessed using different doses of Fgfs, and the involvement of peroxisome proliferator-activated receptor γ (PPARγ) signaling in these processes was tested by introducing a PPARγ agonist (Rosiglitazone) and antagonist (T0070907) to the culture. The expression patterns of Fgf7 and Fgf10 were examined via in situ hybridization of AdBTs.

**Results:**

AdBOs showed higher expression of undifferentiated cell markers and lower expression of differentiated cell markers than AdBTs, NeoBTs, and AdBEs, indicating the relatively immature state of AdBOs. Differentiation of AdBOs was enhanced by Rosiglitazone and Fgf7, suggesting an interplay of intracellular signals between Fgf7 and PPARγ. Co-addition of T0070907 suppressed Fgf7-mediated differentiation, demonstrating that PPARγ is activated downstream of Fgf7 to promote cellular differentiation into umbrella cells. Furthermore, we found that Fgf7 is predominantly expressed in the umbrella cells of the urothelium, whereas Fgf10 is predominantly expressed in the urothelium and stroma of AdBTs.

**Conclusions:**

We demonstrated that unlike Fgf10, Fgf7 induces cellular differentiation via PPARγ activity and has a unique tissue distribution pattern in the adult bladder. Further studies on the Fgf7-PPARγ signaling axis would provide insights into the differentiation mechanisms toward functional umbrella cells and the pathogenesis of several urinary tract diseases.

**Supplementary Information:**

The online version contains supplementary material available at 10.1186/s12894-023-01338-y.

## Background

The urothelium of the bladder is composed of stratified epithelia, which adapt to changes in urine volume and bladder pressure [1]. The urothelium is formed of three types of cells from the basal to the surface layers: keratin 5 (Krt5)-positive undifferentiated basal cells, tumor protein p63 (Trp63)-positive intermediate cells, and uroplakin (Upk)- and keratin 20 (Krt20)-positive terminally differentiated umbrella cells [2]. The basal-to-surface differentiation of the urothelial cells has been reported to be regulated via several signaling pathways, such as peroxisome proliferator-activated receptor γ (PPARγ), sonic hedgehog, arginyl-tRNA synthetase, and Grhl3 [3]. Additionally, failure of terminal differentiation or maturation of urothelial cells, mediated by combinatorial and integrative processes of these multiple signaling cascades, is known to be associated with the development and pathogenesis of several urinary tract disorders [4]. For instance, Upk, a family of genes specifically expressed in terminally differentiated umbrella cells, plays a role in the regulation of urothelial permeability and prevents invasion of pathogens into the bladder tissue [5]. Lee et al. reported that gene knockout of Upk II or Upk IIIa in mice induces nonvoiding contractions or increased micturition pressure of the bladder, thereby resulting in morphological abnormalities as well as dysfunctional voiding, such as an overactive bladder [5]. Moreover, knockout mice of UpkIb, UpkII, or UpkIIIa exhibited improper placement of umbrella cells and developed hydronephrosis, duplication of the pelvis and ureter, and vesicoureteral reflux by morphological transformation of urinary tract tissue, including the ureteric orifice [6, 7]. Thus, it is pertinent to study the mechanisms of stepwise differentiation of immature basal cells toward terminally differentiated umbrella cells in the bladder urothelium to elucidate the pathogenesis of urinary tract disorders caused by urothelial dysfunction.

In recent years, organoid culture systems of the bladder that can maintain both murine- and human-derived healthy and cancerous tissues as three-dimensional structures have been explored as powerful tools for studying the bladder urothelium [8–10]. Among the various approaches that have been published, the organoid model introduced by Mullenders et al. [9] is considered unique, as it enables to culture the normal bladder urothelium of mice by simply isolating the urothelial components via enzymatic digestion of the whole bladder tissue. The authors of that study demonstrated that supplementation with fibroblast growth factor (Fgf)7, Fgf10, and an inhibitor of TGFβ signaling (A83-01) is essential for the growth of organoids established through such a protocol. Despite the simple and easy-to-handle nature of organoid culture systems, the effects of the co-addition of Fgf7 and Fgf10 on the differentiation of bladder urothelium still remain unclear. Both Fgf7 and Fgf10, which are members of the Fgf family, bind to an identical cell surface receptor known as Fgf receptor 2b (*Fgfr2b*) and are believed to exert similar effects on target cells [11]. Moreover, both Fgf7 and Fgf10 have been shown to stimulate growth of the bladder urothelium [12, 13]. It has been suggested that these factors induce overlapping yet distinct intracellular signals owing to quantitatively and qualitatively different levels of Fgfr2b activation, thereby leading to variable stability of the Fgf-Fgfr complex on the cell surface [14]. Additionally, Fgf7 and Fgf10 are produced from different source cell types at certain sites during specific phases [14, 15], allowing these factors to play different roles at the tissue level. In this study, we analyzed the differentiation status of the adult mouse-derived bladder urothelium maintained as organoids. Furthermore, we demonstrated that terminal differentiation of the adult mouse bladder urothelium is promoted by PPARγ signaling, which is activated downstream upon stimulation with Fgf7 but not with Fgf10.

## Materials and methods

### Mice

All the mice used in this study were obtained by mating C57BL/6 mice (CLEA Japan. Inc, Japan) housed in a specific pathogen-free animal room and exposed to a 12:12 h light–dark cycle, under 20–25℃ and 40–60% of humidity. Adult mice aged 8–10 weeks were used for immediate RNA extraction, histological and in situ hybridization (ISH) analyses, and organoid studies. Pups at postnatal day 1 (P1) were used for RNA extraction, followed by gene expression analysis. The number of animals were chosen to ensure obtainment of objective and reliable results, but at the same time minimize the number of animals used. We randomly chose mice for each tissue, molecular, and culture experiments manually in the animal facility. Further, each organoid wells were randomized after treatment with conditioned dosages of Fgf7, Fgf10, DMSO, Ros, and T007 at a different time respectively. Mice were euthanized in CO_2_ gas by experienced personnel. For each animal handling and experiments in vitro, two different investigators were involved as follows: a first investigator (KS) carried out the animal handling, tissue process, and culture experiments. A second investigator (YM) was responsible for subsets of the data analyses and quantification. All animal experiments were approved by the Institutional Animal Care and Use Committee of Juntendo University (registration number:1429; permission number:2,021,155).

### Mouse bladder organoid culture

Bladder organoids were cultured from the bladder specimens of adult C57BL/6 mice, as reported previously [9], with several modifications. Briefly, the whole bladder tissue was minced, placed in a collagenase solution (2 mg/mL of collagenase from Clostridium histolyticum, Sigma-Aldrich, UK, C9891) in Adv DMEM/F-12 (Sigma-Aldrich, UK, 12,634,010), and incubated at 37 °C for 30 min with agitation to liberate the urothelium. After the cell suspension was passed through a 70-μm filter (Corning, USA, 352,350) to remove undigested materials, adult bladder epithelia (AdBE) were collected and placed in individual wells of a prewarmed 24-well plate containing a drop of 35 μL of Matrigel (Corning, USA, 356,231). For culturing adult bladder organoids (AdBOs), the cells in each well were overlaid with 350 μL of Adv DMEM/F-12 medium containing 25 ng/mL of Fgf7 (Peprotech, USA, 100 − 19), 100 ng/mL of Fgf10 (Peprotech, USA, 100 − 26), and 500 nM of A83-01, which is an inhibitor of TGFβ signaling (Tocris Bioscience, UK, 2939). In this study, we used this culture medium as an Fgf7+/Fgf10 + medium to establish bladder organoids from tissue specimens. The medium was changed every three days. Successful organoid culture was defined by 10 individuals in each well > 100 μm of the size within 7 days after culture induction.

Subsequently, Rosiglitazone (Ros, Sigma-Aldrich, USA, R2408), an agonist of PPARγ, or T0070907 (T007, Sigma-Aldrich, USA, T8703), an antagonist of PPARγ, was dissolved in DMSO (Duchefa Biochemie B.V., Netherlands, D1370.0100) and applied to the organoid culture at a concentration of 1 μM. In some experiments, we examined the behavior of organoids cultured in media containing different doses of Fgf7 and Fgf10. In these experiments, the media containing either of the two factors were indicated as Fgf7+/Fgf10- and Fgf7-/Fgf10+. Furthermore, we denoted these media containing four times higher Fgf concentrations, 100 ng/mL of Fgf7 or 400 ng/mL of Fgf10, as 4xFgf7+/Fgf10- or Fgf7-/4xFgf10+, respectively.

### Trypan blue assay

Cell viability was assessed using the Trypan blue assay. The cells were trypsinized using TrypLE™ Express Enzyme (Thermo Fisher Scientific, USA, 12,605,010) at 37 °C for 5 min. The cells were resuspended in 1 mL of PBS (Nacalai Tesque Inc., Japan, 27575-31) containing 1% FBS (Thermo Fisher Scientific, USA, 10270-106) to stop the enzyme reaction. One milliliter of 0.5% trypan blue dye solution (Nacalai Tesque Inc., Japan, 29853-34) was added, the cells were immediately loaded on a hemocytometer (Corning, USA, 480,200), and the number of viable cells per 1 × 1 mm square was counted under a stereo microscope.

### RNA extraction and quantitative realtime (qRT)PCR

RNA extraction and qPCR were performed as previously described [16]. After the animals were anesthetized with isoflurane (2.5%), total RNA was extracted from the indicated specimens (minced whole bladder tissues obtained from adult or P1 mice, collagenase-treated urothelium-enriched cells, or cultured organoids) using a RNeasy Mini Kit (Qiagen, Germany, 74106). Aliquots of 300 ng of total RNA were used for cDNA synthesis with Transcriptor Universal cDNA Master (Roche, Switzerland, 5893151001) in a reaction volume of 20 μL. cDNA (2 μL) was used for qPCR using the Quantitect SYBR Green Kit (Qiagen, Japan, 204145) on a QuantStudio 3 Real-Time PCR System (Thermo Fisher Scientific, USA, A28567). The expression of the genes of interest was normalized to that of the housekeeping gene GAPDH and was calculated using the ΔΔCt method. Sequence of primers (Hokkaido system science co. ltd, Japan) were as follows: Forward 5’ GTCAGGACTGAGGAGAGGGA 3’ and Reverse 5’ TGTCCAGGACCTTGTTCTGC 3’ for Krt5; Forward 5’ CCTCCAACACAGATTACCCG 3’ and Reverse 5’ AGCTTCTTCAGTTCGGTGGA 3’ for Trp63; Forward 5’ AGCGGCAAGAGTGAGATTTCT 3’ and Reverse 5’ CCTCCAGGTTATTCTCCAGGG 3’ for Krt14; Forward 5’ CGAGCACCATCCGAGACTAT 3’ and Reverse primer 5’ TGCAGCCAGCTTAGCATTGT 3’ for Krt20; Forward 5’ GGCCTGACAGCAAAATAATGA 3’ and Reverse 5’ GAGAAGCAGGAAGATGGCTT 3’ for Upk1a; Forward 5’ GTATGGCATCCACACTGCCT 3’ and Reverse 5’ GAGACAGCAGACCAGAGAGG 3’ for Upk2; Forward 5’ AGCGGCTCTTACGAGGTTTA 3’ and Reverse 5’ AGTAGTGCTCAGTGGGACGC 3’ for Upk3a; Forward 5’ CTCTACAGGTCATGCTTCCACC 3’ and Reverse primer 5’ ACAGAACAGTCTTCTCACCCT 3’ for Fgf7; Forward 5’ TTTGGTGTCTTCGTTCCCTGT 3’ and Reverse primer 5’ TAGCTCCGCACATGCCTTC 3’ for Fgf10; Forward 5’ GACATCAAGAAGGTGGTGAAGCAG 3’ and Reverse 5’ ATACCAGGAAATGAGCTTGACAAA 3’ for Gapdh.

### Histological analyses and microscopy

Frozen sections were stained with H&E. The sections were hydrated twice in xylene (Nacalai Tesque Inc., Japan, 36612-35) and 100% ethanol (Nacalai Tesque Inc., Japan, 14713-95); once in 95%, 85%, and 70% ethanol; and twice in deionized H_2_O every 3 min. The samples were stained with hematoxylin (Nacalai Tesque Inc., Japan, 17501-04) for 1 min and eosin for 1 min. Dehydration was performed every 3 min using 70% ethanol, 85% ethanol, 95% ethanol, 100% ethanol, and xylene. Two drops of the mounting reagent was applied, and coverslips were placed onto the slides.

Immunofluorescence was performed on frozen sections as described previously [17]. Following primary antibodies were used according to manufacturer protocols: Cytokeratin 5 (CK5) (EP1601Y, Abcam, UK, ab52635, 1:250) and Uroplakin III (UPK3) (AU1, Progen, Germany, 690,108 S, 1:200). Slides were incubated with primary antibody at 4 °C overnight. Secondary antibody was added in PBS with Tween 20 at room temperature for 1 h at the following dilutions: anti-rabbit Alexa Flour 488 (Invitrogen, USA, A11001), anti-mouse Alexa Flour 594 (Invitrogen, USA, A21468): 1:200 each. Slides were washed with PBS and mounted with coverslips using Vectashield containing DAPI (Vector Laboratories, USA, H-1200). All images were acquired using a BZ-X 710 microscope (KEYENCE, Japan).

### Whole mount immunofluorescence of organoids

Whole mount staining of organoids was performed essentially as described previously [17]. Organoids were fixed in 4% PFA for 30 min followed by treatment with 50 mM NH_4_Cl (Nacalai Tesque Inc., Japan, 02423-45) for 30 min. Permeabilization was performed with 0.1% Triton X-100 (Polysciences, USA, 04605 − 250) for 30 min, and then blocking was performed with 5% bovine serum albumin (Nacalai Tesque Inc., Japan, 01863-51) for 1 h. Thereafter, organoids were incubated with primary antibodies overnight at 4 °C and then with the secondary antibody for 1 h at room temperature. Primary and secondary antibodies were used in the same concentrations described earlier. All samples were treated with Hoechst 33,342 (Tocris Bioscience, UK, 5117) for nuclear staining. Images were acquired using a TCS-SP5 confocal laser scanning microscope (Leica, Germany).

### In situ hybridization (ISH)

ISH for Fgf7 and Fgf10 was performed as described previously [18]. The pcDNA3 plasmid containing a cDNA fragment of mouse Fgf7 (nucleotides 193–769; GenBank accession no. NM008008.4) and Fgf10 (nucleotides 533–1135; GenBank accession No. NM008002.4) was constructed. Single-stranded digoxigenin-labelled RNA probes were generated using an in vitro transcription system (Roche, Switzerland, 11,277,073,910). Frozen sections were rehydrated, treated with HCl (Nacalai Tesque Inc., Japan, 28514-75), digested in Proteinase K solution (Roche, Switzerland, 03115844001), post-fixed, treated in acetic anhydride solution (Nacalai Tesque Inc., Japan, 00211 − 95), and hybridized overnight at 60 and 65 °C with probes for Fgf7 and Fgf10, respectively. After extensive rinsing and washing, the sections were subjected to immunohistochemistry using an alkaline phosphatase–conjugated anti-digoxigenin antibody (Roche, Switzerland, 1,093,274,910). The sections were then treated with nitroblue tetrazolium/5-bromo-4-chloro-3-indolyl phosphate solution (Roche, Switzerland, 11,383,213,001 and 11,383,221,001) for color development. Images were acquired using a microscope (BZ-X710; Keyence).

### Statistical analysis

**Fig. 1:**
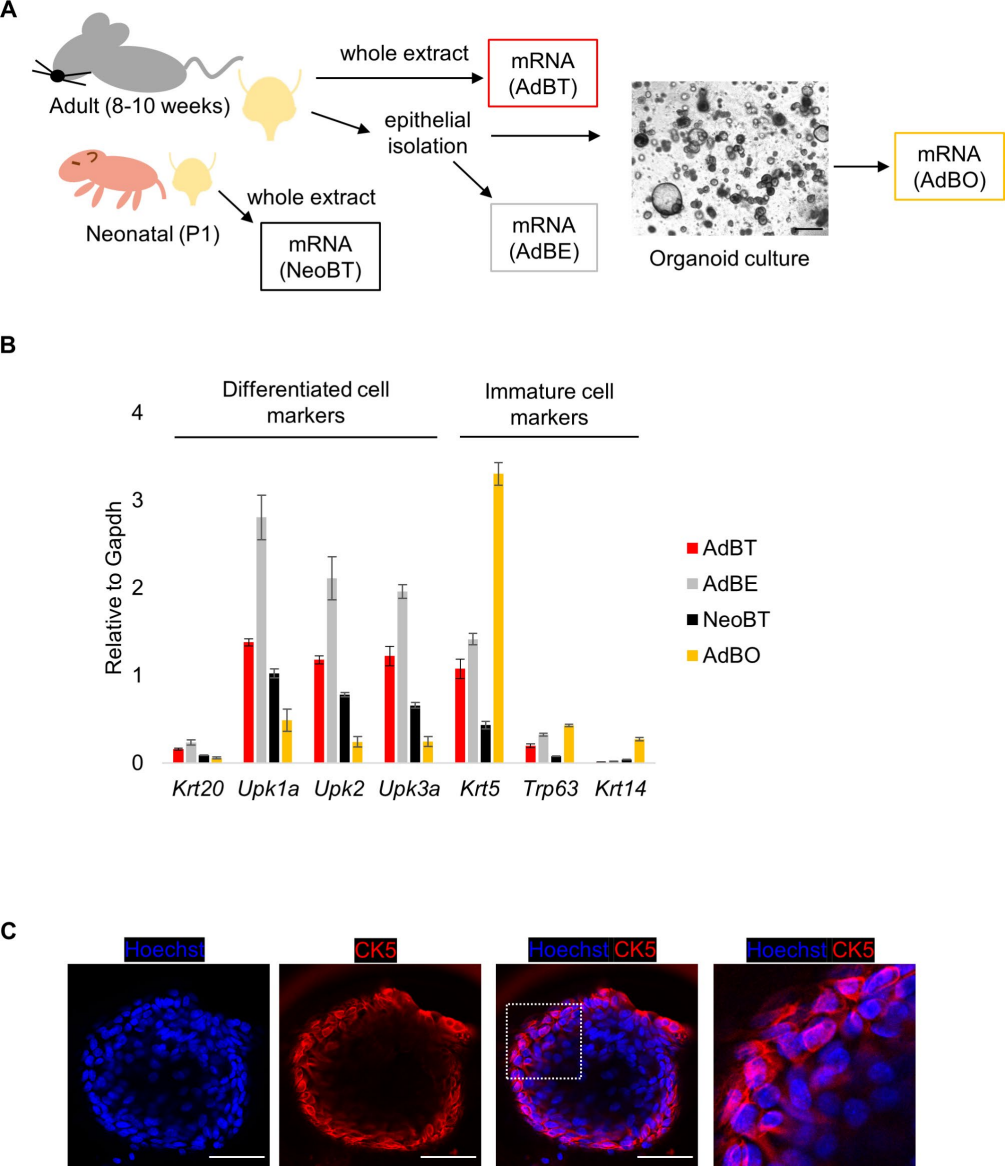
Differentiation status of bladder organoid derived from adult mice. (**A**) Schematic representation of the experiment. Bladders were taken out from adult (8–10 weeks of age) or neonatal mice (postnatal day 1). Adult bladders were further processed for epithelial isolation for following organoid culture. mRNA were isolated from the whole extract of adult (adult bladder tissues; AdBT) bladders, neonatal (neonatal bladder tissues; NeoBT) bladders, the isolated epithelia (adult bladder epithelium; AdBE), and organoid cells cultured for 1 week (adult bladder organoids; AdBO) for qPCR. A representative image of AdBO is shown. Scale bar: 500 μm. (**B**) Expression levels of the differentiated cell markers (Krt20, Upk1a, Upk2, Upk3a) and immature cell markers (Krt5, Krt14, Trp63) in AdBT, AdBE, NeoBT, and AdBO as values adjusted with Gapdh levels are graphed by qPCR, respectively. Values represent the average of three independent experiments and error bars denote standard error of the mean. (**C**) Immunofluorescence of murine bladder organoids for staining with CK5 shown with Hoechst. Scale bar: 50 μm

**Fig. 2:**
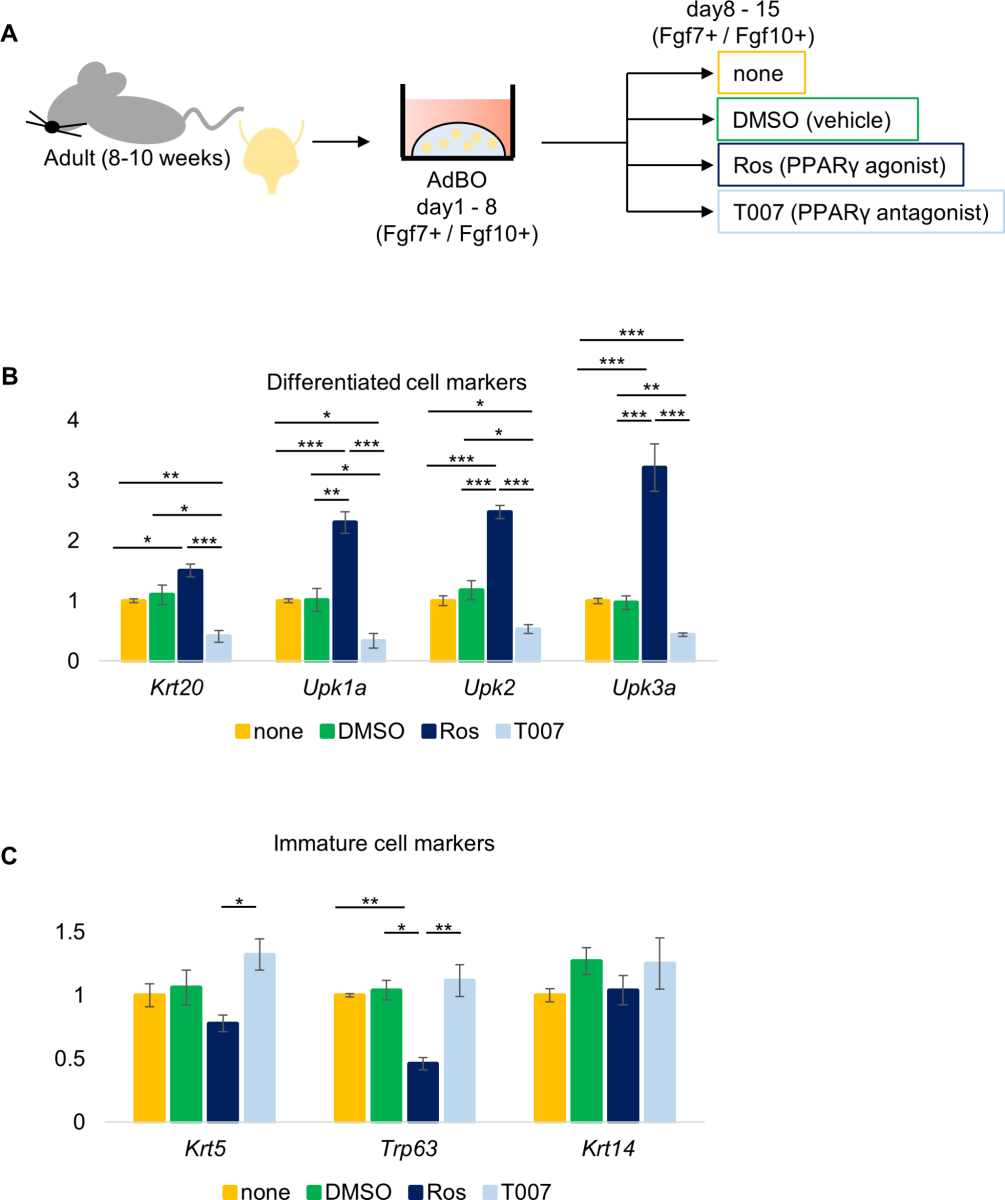
The effect of PPARγ signal on differentiation of bladder organoids. (**A**) Experimental protocol. AdBOs were grown for 1 week (Day 1 to 8), split to multiple wells, and divided into 4 groups. Each group of organoids was then treated with vehicle alone (DMSO), 1 μM Ros, 1 μM T007, or untreated (none) (Day 8 to 15). After 1 week of treatment, mRNA were isolated and subjected for qPCR on Day 15. (**B**) and (**C**) Relative expression levels of the differentiated cell markers (Krt20, Upk1a, Upk2, Upk3a) (**B**) and immature cell markers (Krt5, Trp63, Krt14) (**C**) in 4 groups of organoids. Values represent the average of three independent experiments and error bars denote standard error of the mean. ***p < 0.001, **p < 0.01 and *p < 0.05

**Fig. 3:**
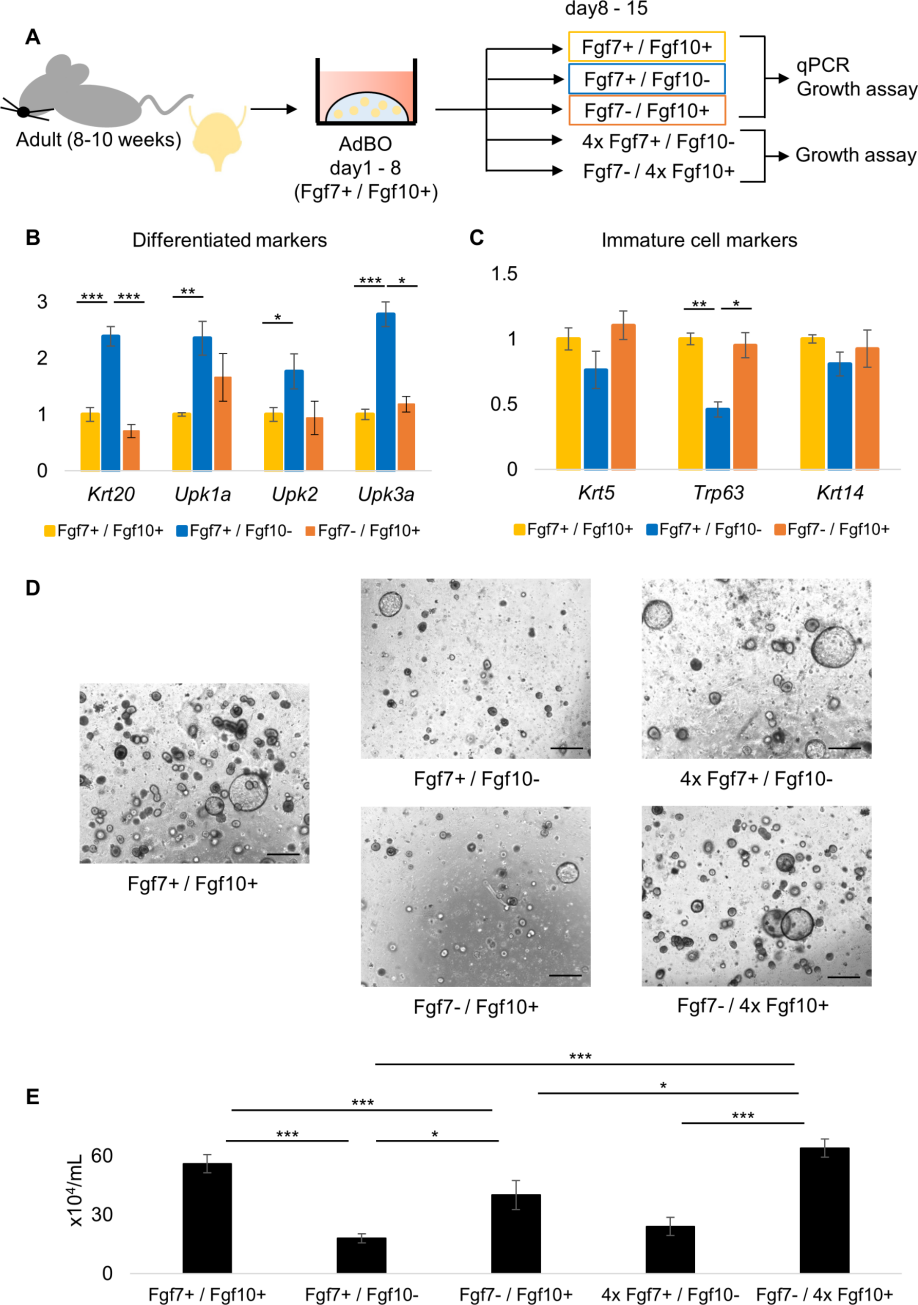
Fgf7 but Fgf10 induces differentiation of organoid cells. (**A**) Experimental protocol. AdBOs were grown for the first week (Day 1 to 8) in Fgf7+/Fgf10 + medium, split to multiple wells, and divided into 5 groups. Each group of organoids was cultured in Fgf7+/Fgf10+, Fgf7+/Fgf10-, Fgf7-/Fgf10+, 4xFgf7+/Fgf10-, or Fgf7-/4xFgf10 + medium for additional 1 week (Day 8 to 15) and then used for the following assays as indicated. (**B**) and (**C**) Relative expression levels of differentiated cell marker genes (Krt20, Upk1a, Upk2, Upk3a) (**B**) and immature cell marker genes (Krt5, Trp63, Krt14) (**C**) in organoids cultured in Fgf7+/Fgf10+, Fgf7+/Fgf10-, or Fgf7-/Fgf10 + medium. ***p < 0.001, **p < 0.01 and *p < 0.05. (**D**) AdBOs were grown for 1 week (Day 1 to 8) in Fgf7+/Fgf10 + medium and then cultured under 5 different conditions for additional 1 week (Day 8 to 15). Representative images of organoids in each group acquired on Day 15 were shown. Scale bar: 500 μm. (E) The number of viable cells in AdBOs cultured under 5 different conditions was assessed on Day 15 by Trypan blue exclusion assay. Values represent the average of three independent experiments and error bars denote standard error of the mean. ***p < 0.001, **p < 0.01 and *p < 0.05

**Fig. 4:**
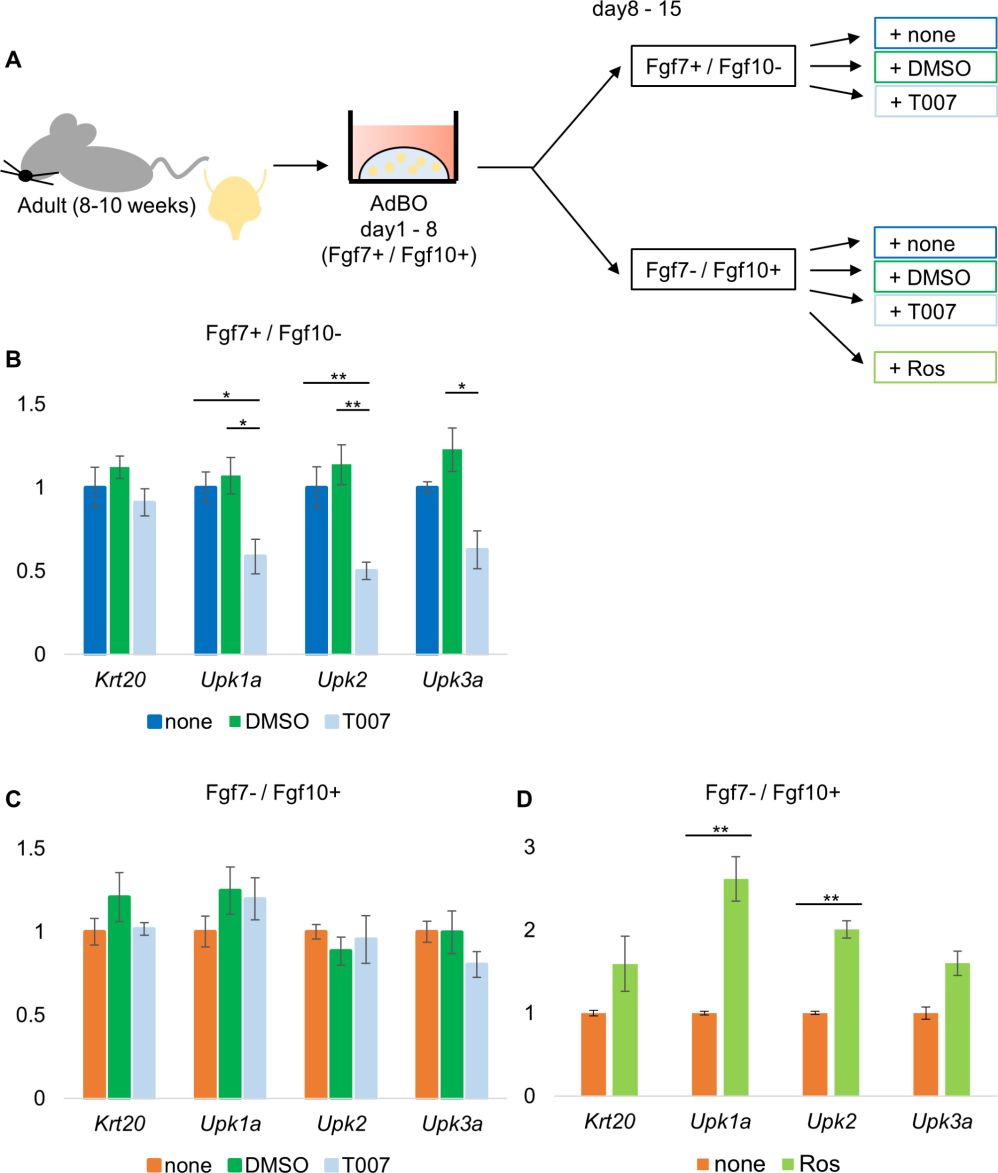
Fgf7-PPARγ axis has a pivotal role to induce differentiation in bladder organoids. (**A**) Experimental protocol. AdBOs were grown for 1 week (Day 1 to 8) in Fgf7+/Fgf10 + medium, split to multiple wells, and divided into 6 groups. Three out of 6 groups of organoids were cultured in Fgf7+/Fgf10- medium and the other three were in Fgf7-/Fgf10 + medium, respectively. Then each of three groups was treated with vehicle alone (DMSO), 1 μM T007, or untreated (none) for the next 1 week (Day 8 to 15). Additionally, 1 μM Ros was added only in Fgf7-/Fgf10 + medium. mRNA were isolated and subjected for qPCR on Day 15. (**B**) and (**C**) Relative expression levels of differentiated cell marker genes (Krt20, Upk1a, Upk2, Upk3a) in organoids cultured in the presence of Fgf7 (Fgf7+/Fgf10-) (**B**) or Fgf10 (Fgf7-/Fgf10+) (**C**). Values represent the average of three independent experiments and error bars denote standard error of the mean. **p < 0.01 and *p < 0.05

The number of pups at P1 used for gene expression analysis was three (NeoBT, n = 3). Regarding the adult mice, the first group of 3 mice were used for ISH and gene analyses presented in Fig. 1B C, and 5C. After isolation of the bladder, each of three was divided into four pieces. One is fixed and used for ISH (n = 3). The second piece was used for mRNA isolation (AdBT, n = 3) and gene analysis. The third and fourth pieces were used for epithelial isolation and used for direct mRNA isolation (AdBE, n = 3) and organoid culture that were used for gene analysis and whole mount staining at later time points (AdBO, n = 3), respectively. Another group of 3 mice were used for organoid culture and gene analyses shown in Figs. 2 and 4 (n = 3). The last group of 3 mice were used for organoid culture, growth assay, and gene analyses presented in Fig. 3). We selected a small sample size because the murine blader organoid phenotype treated by PPARγ was evaluated in vitro for the first time in the present study, and therefore, the initial intention was to gather evidence regarding the biological effect in the current experimental designs.

Differences between the two groups were tested using an unpaired t-test. All statistical tests were two-sided. All data were expressed as mean ± standard error of the mean (SEM). Statistical significance was set at P < 0.05.

## Results

### Analysis of differentiation status in bladder organoid cells

To investigate how culturing murine urothelial cells as organoids changes their differentiation status, we compared the expression levels of several genes in AdBOs with those in adult bladder tissues (AdBTs) and neonatal bladder tissues (NeoBT). AdBOs were cultured in medium containing Fgf7, Fgf10, and A83-01 (Fgf7+ / Fgf10 + medium), as previously described [9]. mRNA was extracted from AdBOs cultured for one week or from AdBTs and NeoBTs immediately after bladder tissue excision and examined via qPCR (Fig. 1A). The relative expression levels of Krt20, Upk1a, Upk2, and Upk3a, which are well-known marker genes for differentiated cell types, were lower in AdBO than in AdBT or NeoBT when adjusted with the level of Gapdh expression (Fig. 1B and Supplementary Fig. 1).

In contrast, marker genes for undifferentiated immature cell types, such as Krt5, a gene abundantly present in basal and intermediate cells, and Trp63, a representative marker gene for intermediate cells, were expressed at higher levels in AdBO than in others (Fig. 1C).

In contrast, marker genes for undifferentiated immature cell types, such as Krt5, a gene abundantly present in basal and intermediate cells, and Trp63, a representative marker gene for intermediate cells, were expressed at higher levels in AdBO than in others (Fig. 1B and Supplementary Fig. 1).

As maturation of the bladder urothelium to its adult phenotype occurs even after birth, urothelial cells in the early postnatal phases are known to be different from those in adults in terms of gene expression profiles. For example, Krt14 is activated in the premature postnatal urothelium and is thus highly expressed in cells in NeoBT compared to those in AdBT [19]. Consistently, Krt14 expression was higher in NeoBT than in AdBT in our qPCR analysis (Fig. 1B). Importantly, the level of Krt14 was significantly higher in AdBO than in AdBT and NeoBT, suggesting a unique premature feature of cells in AdBO (Fig. 1B). To exclude the possibility that enrichment of the urothelium in AdBO due to removal of non-epithelial components might affect the relative analyses in our qPCR data, we compared the expression levels of genes in AdBO and AdBE, a urothelium-rich population obtained after digesting whole bladder tissues with collagenase (Fig. 1B). However, consistent with the previous results, qPCR showed lower expression levels of Krt20, Upk1a, Upk2, and Upk3a (Fig. 1B) but higher levels of Krt5, Trp63, and Krt14 (Fig. 1B) in AdBO than in AdBE. Although we could not perform a similar direct comparison between neonatal bladder epithelia and AdBO owing to the difficulty in isolating small neonatal urothelia, our data showed that the culture conditions for AdBO induced a shift of urothelial cells toward an immature status, which allowed for intense expression of Krt14 whose expression is relatively high in the neonatal urothelium.

Whole mount immunofluorescence of AdBOs further supported this notion. We found that AdBOs were composed of multi-layered structure as judged by nuclear staining with Hoechst 33,342, and contained abundant CK5-positive cells, strongly supporting the immature feature of AdBOs (Fig. 1C).

### The effect of PPARγ signaling on the differentiation of bladder organoids

PPARγ, a nuclear receptor-type transcription factor, regulates target gene expression by binding to peroxisome proliferator response elements as a heterodimer with Retinoid X Receptor α [20, 21]. It was shown previously that in the bladder, PPARγ regulates the expression of Upk genes (a member of the transmembrane 4 superfamily) in terminally differentiated umbrella cells, which play a major role in the urothelial barrier function at the inner-most surface [2, 20]. Therefore, we tested whether PPARγ promotes bladder epithelial differentiation in AdBO. We cultured AdBO in Fgf7+ / Fgf10 + medium for the first week (from day 1 to 8), split them into four groups, treated them with either vehicle (DMSO), a PPARγ agonist (Ros), or its antagonist (T007), or left them untreated (none) for another week (from day 8 to 15) and then assessed the differentiation status of cells via qPCR (Fig. 2A). Ros-mediated activation of PPARγ resulted in the increased expression of Krt20, Upk1a, Upk2, and Upk3a (Fig. 2B and Supplementary Fig. 2). In contrast, T007 treatment led to a decrease in these differentiated cell markers (Fig. 2B and Supplementary Fig. 2). Regarding immature cell marker genes, only the expression of Trp63 was downregulated by Ros, and neither Ros nor T007 induced significant changes in the expression levels of Krt5 or Krt14 (Fig. 2C and Supplementary Fig. 2). Together, our data was consistent with the previous finding that PPARγ signaling promotes terminal differentiation of urothelial cells. Our data obtained using T007 also suggested the presence of basal levels of PPARγ activity in AdBO maintained in the Fgf7+ / Fgf10 + medium.

### Fgf7 induces differentiation of organoid cells

The original report of the bladder organoid system employed in the present study utilized Fgf7 and Fgf10 as essential growth factor supplements without exploring their distinct activities [9]. Next, we investigated how Fgf7 and Fgf10, known to bind to identical cell surface receptors to transmit signals, may be associated with the differentiation and proliferation of cells in AdBO in culture. After a primary culture of AdBO in Fgf7+ / Fgf10 + medium for a week (from day 1 to 8), organoids were divided into three groups for testing in either Fgf7+ / Fgf10+, Fgf7+ / Fgf10-, or Fgf7- / Fgf10 + media for another week (from day 8 to 15), and then subjected to gene analysis by qPCR (Fig. 3A). The expression levels of Krt20, Upk1a, Upk2, and Upk3 were significantly higher in cells cultured with Fgf7+ / Fgf10- than in those cultured under the other two conditions (Fig. 3B). We also found that, when cultured with Fgf7- / Fgf10+, expression of Krt20 was downregulated compared to that in cells cultured with Fgf7+ / Fgf10- (Fig. 3B). Regarding immature cell markers, the expression of Trp63 was decreased in Fgf7+ / Fgf10- medium compared to that in Fgf7+ / Fgf10+ (Fig. 3C). Krt5 and Krt14 did not show any detectable differences among the three groups (Fig. 3C). These data suggest that Fgf7, but not Fgf10, upregulates the expression of representative terminal differentiation marker genes of the urothelium in AdBO.

Next, we assessed the effect of Fgf7 and Fgf10 on AdBO cell proliferation using a trypan blue dye exclusion growth assay. When we analyzed three culture conditions as tested for organoid differentiation (Fig. 3A), AdBO cultured with Fgf7+ / Fgf10 + showed a higher proliferation rate than those cultured with Fgf7+ / Fgf10- or Fgf7- / Fgf10+, suggesting that Fgf7 and Fgf10 have growth-supportive activity and have additive effects. In order to see more clearly the effect of each factor on organoid growth, we also performed growth assays with AdBO cultured with 4 times higher dose of Fgf7 (4x Fgf7+ / Fgf10- medium) or Fgf10 (Fgf7- / 4x Fgf10 + medium). AdBOs cultured with Fgf7- / 4x Fgf10 + medium showed higher growth rate compared to that of cells cultured with Fgf7- / Fgf10+, demonstrating that Fgf10 had a dose-dependent growth-promoting effect (Fig. 3D, E). Meanwhile, AdBOs in 4x Fgf7+ / Fgf10- medium stayed as proliferative as those in Fgf7+ / Fgf10-, suggesting that the effect of Fgf7 on cell proliferation is not potent as that of Fgf10. Taken together, our data showed that Fgf7 and Fgf10 have overlapping but distinct effects on AdBO; they both have certain levels of growth-promoting effects, but only Fgf7 shows a discernible function in inducing terminal differentiation of AdBO. Furthermore, our observation that the genes upregulated by Fgf7 were transcriptionally activated by Ros raised the possibility that there might be a functional relationship between intracellular signaling triggered by Fgf7 and PPARγ-mediated gene transcription.

### Fgf7-PPARγ axis has a pivotal role to induce differentiation in bladder organoids

We analyzed whether PPARγ activity was regulated downstream of Fgf7 in AdBO. We grew AdBO in Fgf7+ / Fgf10 + medium for one week (from days 1 to 8), split them, and then cultured them in the presence of only Fgf7 but not Fgf10 (Fgf7+ / Fgf10- medium) for another week (from days 8 to 15). They were treated with vehicle alone (DMSO), T007, or left untreated (none) and subjected to gene expression analysis (Fig. 4A). We also performed similar experiments to test the inhibitory effect of T007 on Fgf10 signaling, while maintaining AdBO in the presence of only Fgf10 (Fgf7- / Fgf10 + medium) (Fig. 4A). As shown in Fig. 4B, co-addition of T007 with Fgf7 decreased the mRNA levels of Upk1a, Upk2, and Upk3a but not Krt20, indicating that PPARγ activity is controlled downstream of Fgf7 signaling in AdBO (Fig. 4B). In contrast, no significant change in differentiated cell markers was observed when T007 was supplemented in the AdBO culture with Fgf7- / Fgf10+, indicating that PPARγ-mediated transcription of these genes in AdBO was not activated by Fgf10 (Fig. 4C). We further compared the expression levels of differentiated cell markers in AdBOs cultured with Fgf7- / Fgf10 + medium plus Ros, with those cultured with only Fgf10 (Fgf7- / Fgf10+) (Fig. 4A). The expression of those markers was elevated in the presence of Ros, indicating that a certain fraction of PPARg in AdBOs are poised for transcriptional activation independently of Fgf7 (Fig. 4D). These results may suggest that Fgf7 is not an essential factor to activate PPARγ but a modulator to intensify its activity, for example through enhancing its ligand binding affinity, nuclear translocation efficiency, transcriptional activation in the nucleus, and so on. Collectively, these data suggest that both Fgf7 and Fgf10 stimulate cellular proliferation in AdBO, whereas only Fgf7 affects cellular differentiation toward umbrella cell types via the activation of PPARγ through yet unknown mechanisms.

### In situ hybridization for Fgf7 and Fgf10 of murine bladder tissue

**Fig. 5:**
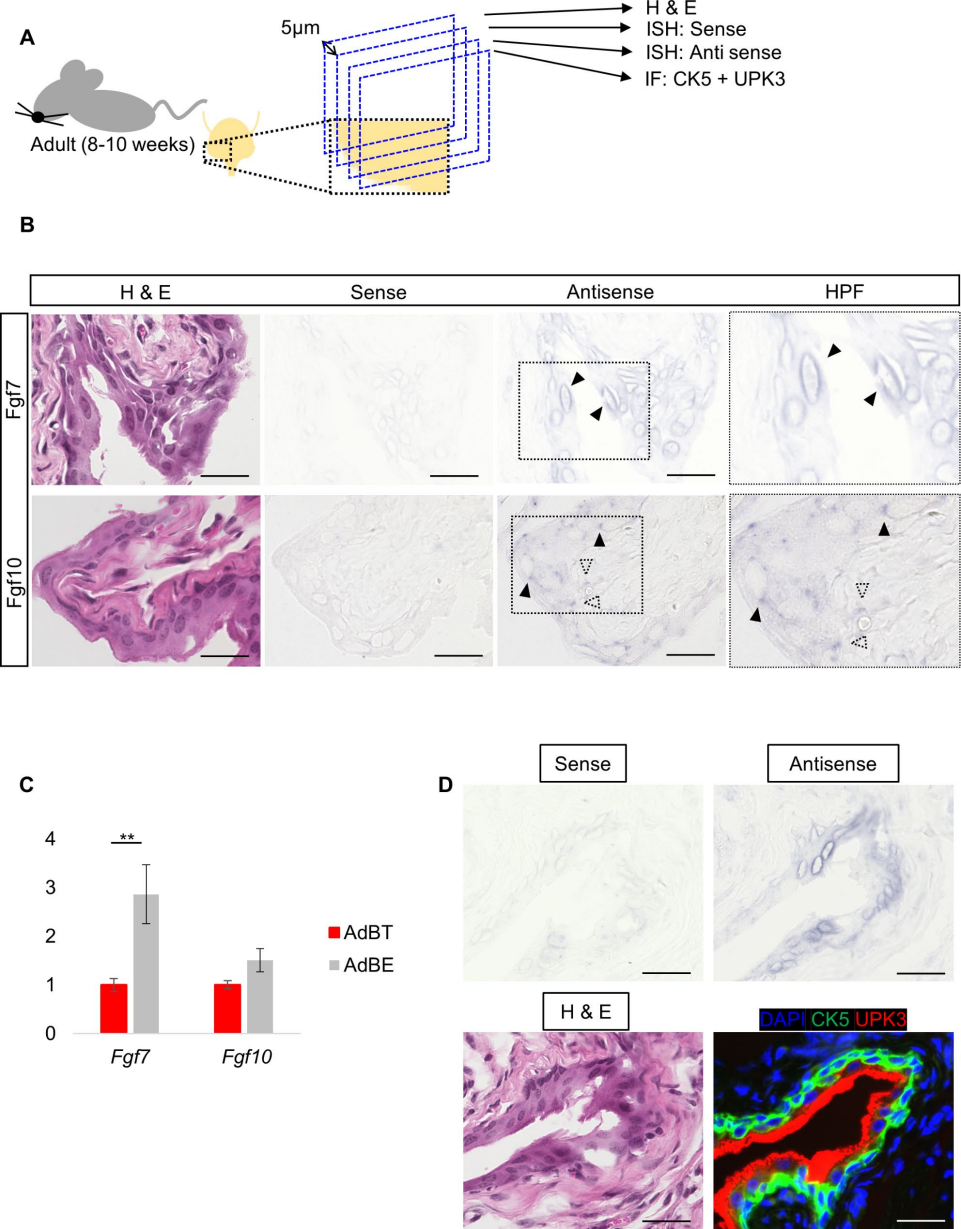
Expression patterns of Fgf7 and Fgf10 mRNA in murine bladder tissues. (**A**) Experimental protocol. The adult murine bladder specimens were sectioned at 5 mm and three serial sections were used for H&E staining, in-situ hybridization (ISH) with sense probes, or ISH with antisense probes, and immunofluorescence for CK5 and UPK3 staining. (**B**) Representative images for Fgf7 (top) and Fgf10 (bottom) analyses are shown. Histological image (H&E), ISH images with sense and anti-sense probes are shown from left to right. The magnified views from the dashed rectangles are shown in the rightmost column. Arrowheads and dotted arrowheads indicate signals of antisense at epithelial and stroma layer, respectively. Scale bar: 25 μm. (**C**) Relative expression levels of Fgf7 and Fgf10 in AdBT and AdBE by qPCR. **p < 0.01. (**D**) Other representative images obtained from different individual from B) for Fgf7 analysis are shown. ISH images with sense and anti-sense probes (upper left and right) and Histological images for H&E (lower left) and complementary staining with CK5 and UPK3 (lower right) are shown. Scale bar: 25 μm

Three major cell types, basal cells, intermediate cells, and umbrella cells, form stratified layers from the basal to luminal side and constitute the bladder urothelium. Compared with basal or intermediate cells, umbrella cells are characterized by a large cytoplasm with relatively large nuclei [22]. Based on our observation that Fgf7, but not Fgf10, is involved in cellular differentiation toward umbrella cells, we were interested in the expression patterns of these two factors in AdBT and therefore conducted ISH (Fig. 5A). To precisely locate cells that stained positively, ISH using sense and antisense probes for each gene was performed using serial sections cut at 5 μm thickness with reference to adjacent sections stained for H&E (Fig. 5A).

Fgf7 mRNA was broadly detected in the urothelium and was indiscernible in the underlying mesenchymal tissues (Fig. 5B, top). In contrast to Fgf7, expression of Fgf10 was observed in both the urothelium and stroma without any noticeable association with particular cell types (Fig. 5B, bottom). In order to see such different tissue distribution of Fgf7 and Fgf10 by an alternative method, we examined mRNA expression of these two factors in AdBT and AdBE by qPCR. It was revealed that Fgf7 mRNA was more abundant in AdBE than in AdBT, supporting the uneven distribution of Fgf7 in the adult urothelium (Fig. 5C). In contrast, the mRNA levels of Fgf10 did not show significant difference between AdBT and AdBE, suggesting that the distribution of Fgf10 in the bladder tissues is not skewed as that of Fgf7 between the urothelium and the stroma (Fig. 5C).

Interestingly, we noticed in our ISH that, in the urothelial layers, Fgf7 signals were mostly observed at the periphery of large oval nuclei [22], which is a characteristic feature of umbrella cells located at the luminal surface (Fig. 5B, top). To further examine the specific localization of Fgf7-expressing cells, we conducted an additional ISH assay, together with complementary immunofluorescence of CK5 and UPK3, by using another set of serial sections (Fig. 5A). We found that Fgf7 mRNA was predominantly detected in the surface epithelial cells that were UPK3 + but CK5- (Fig. 5D). It was also demonstrated that epithelial cells in more basal layers, which were UPK3- and CK5+, contain less Fgf7 + cells than the surface epithelial cells (Fig. 5D).

These data revealed that their source cell types are distinct and that Fgf7 is produced predominantly in the urothelium compared to Fgf10. Our data also suggest that Fgf7 is expressed as a function of cells located in the vicinity of the surface of the urothelium, mainly composed of terminally differentiated umbrella cells.

## Discussion

In the present study, we demonstrated that Fgf7 and Fgf10 act distinctively on cell differentiation of adult mouse-derived bladder urothelium: Fgf7, but Fgf10, induces terminal differentiation of organoid cells toward umbrella cells. There are several reports regarding the roles of Fgf7 signaling in the proliferation of the bladder urothelium. Tash et al. reported that Fgf7 is essential for the proliferation of intermediate cells, which leads to the formation of a multilayered structure of the urothelium using conventional Fgf7 KO mice [23]. Another study by Narla et al. showed that Fgf7-mediated urothelial proliferation contributes to tissue regeneration after damage induced by cyclophosphamide in a mouse model [24]. In these studies, Fgf7 was shown to promote cellular proliferation, which led to an expansion of cell populations and a resultant increase in the supply of terminally differentiated cells in the bladder urothelium. Thus, to the best of our knowledge, our study is the first to directly demonstrate that Fgf7 plays a role in the mechanism of terminal differentiation of bladder urothelial cells. We showed that Fgf7 upregulates mRNA levels of Krt20, Upk1a, Upk1, and Upk3, all of which are marker genes for umbrella cells in organoid cultures. Previous findings that such marker genes for umbrella cell types can be direct targets of PPARγ-mediated transcription [10, 20] led us to conceive a possible functional interaction between Fgf7 and PPARγ signals. Remarkably, addition of the PPARγ agonist (Ros) upregulated the mRNA expression of the same gene set in our organoid culture system as Fgf7 did. In addition, our observation that enhanced expression of Upk1a, Upk2, and Upk3 in Fgf7+ / Fgf10- medium was suppressed by the PPARγ antagonist (T007) clearly showed that PPARγ activates differentiation genes downstream of the Fgf7 signal.

Interestingly, increases in the differentiation marker genes of umbrella cells in response to Fgf7 were accompanied by the relative downregulation of Trp63, a marker gene for intermediate cells, but not by Krt5, a basal cell marker. This implies that among several phases toward urothelial terminal differentiation, the Fgf7-PPARγ axis could be predominantly involved in the induction of late phase differentiation from intermediate to umbrella cells, rather than the early phase from basal to intermediate cells. Further studies on the detailed mechanisms of terminal differentiation induced by the Fgf7-PPARγ signaling axis would be important to understand how well-coordinated stepwise differentiation of the bladder urothelium is regulated.

Notably, Fgf10 acted differently compared to Fgf7: organoids cultured with Fgf7- / Fgf10 + medium did not induce differentiation or interact with PPARγ signaling. A previous study showed that in adipocytes, Fgf10 induces cellular differentiation by consecutive activation of a transcription factor, Krüppel-like factor, and PPARγ [25]. Considering the different roles of Fgf10 in different systems, it is suggested that how Fgf10 activates intracellular signaling pathways and regulates cell fate, including proliferation and differentiation, may depend on tissue and cell type.

In this study, we utilized a basal-type bladder organoid system that uses chopped bladder tissues followed by enzymatic digestion as a starting material and contains immature urothelial cells predominantly [9]. Mullenders et al. also described in their report that if the authors collect urothelia rich for luminally superficial umbrella cells, those populations grow as suprabasal-type bladder organoids [9]. Considering that the culture conditions after urothelial isolation were identical between the two systems, this indicated that the degree of differentiation status of the organoid cells could be dependent on how the cells were prepared before initiating the culture, but not on the dynamic progression of terminal differentiation toward umbrella cells during culture. Using a basal-type organoid culture system, we showed that AdBO cells remain undifferentiated, as they show relatively higher expression of marker genes for undifferentiated phenotypes, such as Krt5 and Trp63, than AdBT cells. Moreover, we found that the expression of Krt14 in AdBO was higher than that in NeoBT, suggesting the acquisition of unexpectedly immature features in AdBO. Although it remains unclear why AdBO reverts to such an immature state in culture, the basal-type organoids used in this study were shown to be an applicable tool for investigating the regulatory mechanism of urothelial differentiation via the Fgf7-PPARγ axis.

In this study, we also demonstrated that the mRNA expression patterns of Fgf7 and Fgf10 were different in the adult mouse bladder. A previous report showed that in mouse embryonic bladder tissues, Fgf7 mRNA was predominantly detected in the stroma [26]. Another study reported that in the human adult bladder, Fgf10 mRNA was expressed in the lamina propria [13]. Thus, our study is the first to show the expression sites of Fgf7 and Fgf10 in the adult mouse bladder. Interestingly, Fgf7 was predominantly detected in the urothelium, whereas Fgf10 was broadly expressed in both epithelial and non-epithelial layers, suggesting that the production sites of Fgf7 and Fgf10 may differ depending on the developmental stage and animal species. One remarkable finding of our study was that Fgf7 expression was predominantly detected in urothelial cells with large oval nuclei, a typical feature of umbrella cells. Together with our data on the in vitro effects of Fgf7, this suggests that Fgf7, but not Fgf10, is secreted by cells that have undergone differentiation and maturation, and then induces cellular differentiation in the surrounding area via autocrine or paracrine mechanisms.

## Conclusion

Collectively, we demonstrated that, by using an organoid system, Fgf7 serves as an inducer of cellular differentiation toward umbrella cells by activating PPARγ. It has been suggested that such functions of Fgf7 and its unique distribution in the mouse adult bladder could be involved in the terminal differentiation process from intermediate to umbrella cells. Further investigation of the mechanism of terminal differentiation of bladder urothelia regulated by Fgf7-PPARγ-axis signaling using organoid culture would be beneficial to better understand the etiopathology of several urinary tract diseases, as well as a strategy to induce differentiation toward functional umbrella cells for tissue regeneration.

### Electronic supplementary material

Below is the link to the electronic supplementary material.

Supplementary Material 1

Supplementary Material 2

## Data Availability

The datasets used during the current study available from the corresponding author on request.

## References

[1] Truschel ST, Wang E, Ruiz WG, Leung SM, Rojas R, Lavelle J (2002). Stretch-regulated exocytosis/endocytosis in bladder umbrella cells. Mol Biol Cell.

[2] Ho PL, Kurtova A, Chan KS (2012). Normal and neoplastic urothelial stem cells: getting to the root of the problem. Nat Rev Urol.

[3] Wiessner GB, Plumber SA, Xiang T, Mendelsohn CL. Development, regeneration and tumorigenesis of the urothelium. Development 2022;149. 10.1242/dev.198184 PMC10656457 35521701

[4] Kątnik-Prastowska I, Lis J, Matejuk A (2014). Glycosylation of uroplakins. Implications for bladder physiopathology. Glycoconj J.

[5] Lee G (2011). Uroplakins in the lower urinary tract. Int Neurourol J.

[6] Wu XR, Kong XP, Pellicer A, Kreibich G, Sun TT (2009). Uroplakins in urothelial biology, function, and Disease. Kidney Int.

[7] Carpenter AR, Becknell MB, Ching CB, Cuaresma EJ, Chen X, Hains DS (2016). Uroplakin 1b is critical in urinary tract development and urothelial differentiation and homeostasis. Kidney Int.

[8] Melzer MK, Zehe V, Zengerling F, Wezel F, Günes C, Maisch P (2022). [Organoids as a milestone on the way to personalized treatment of urothelial carcinoma: a systematic review]. Urologie.

[9] Mullenders J, de Jongh E, Brousali A, Roosen M, Blom JPA, Begthel H (2019). Mouse and human urothelial cancer organoids: a tool for Bladder cancer research. Proc Natl Acad Sci U S A.

[10] Santos CP, Lapi E, Martínez de Villarreal J, Álvaro-Espinosa L, Fernández-Barral A, Barbáchano A (2019). Urothelial organoids originating from CD49fhigh mouse stem cells display notch-dependent differentiation capacity. Nat Commun.

[11] Turner N, Grose R (2010). Fibroblast growth factor signalling: from development to cancer. Nat Rev Cancer.

[12] Bassuk JA, Cochrane K, Mitchell ME (2003). Induction of urothelial cell proliferation by fibroblast growth factor-7 in RAG1-deficient mice. Adv Exp Med Biol.

[13] Bagai S, Rubio E, Cheng JF, Sweet R, Thomas R, Fuchs E (2002). Fibroblast growth factor-10 is a mitogen for urothelial cells. J Biol Chem.

[14] Zinkle A, Mohammadi M (2019). Structural biology of the FGF7 subfamily. Front Genet.

[15] Makarenkova HP, Hoffman MP, Beenken A, Eliseenkova AV, Meech R, Tsau C (2009). Differential interactions of FGFs with heparan sulfate control gradient formation and branching morphogenesis. Sci Signal.

[16] Matsumoto Y, Koga H, Takahashi M, Suda K, Ochi T, Seo S (2021). Defined serum-free culture of human infant small intestinal organoids with predetermined doses of Wnt3a and R-spondin1 from surgical specimens. Pediatr Surg Int.

[17] Suda K, Matsumoto Y, Ochi T, Koga H, Lane GJ, Hattori N, Nakamura T, Yamataka A (2022). Successful engraftment of bladder organoids in de-epithelialized mouse colon. Pediatr Surg Int.

[18] Fukuda M, Mizutani T, Mochizuki W, Matsumoto T, Nozaki K, Sakamaki Y, Ichinose S, Okada Y, Tanaka T, Watanabe M (2014). Small intestinal stem cell identity is maintained with functional paneth cells in heterotopically grafted epithelium onto the colon. Genes Dev.

[19] Papafotiou G, Paraskevopoulou V, Vasilaki E, Kanaki Z, Paschalidis N, Klinakis A (2016). KRT14 marks a subpopulation of bladder basal cells with pivotal role in regeneration and tumorigenesis. Nat Commun.

[20] Liu C, Tate T, Batourina E, Truschel ST, Potter S, Adam M, Xiang T, Picard M, Reiley M, Schneider K (2019). Pparg promotes differentiation and regulates mitochondrial gene expression in bladder epithelial cells. Nat Commun.

[21] Desvergne B, Wahli W (1999). Peroxisome proliferator-activated receptors: nuclear control of metabolism. Endocr Rev.

[22] Palaoro LA, Guerra F, Angeleri A, Palamas M, Melba SS, Rocher AE (2012). Urothelial cells in smears from cervix uteri. J Cytol.

[23] Tash JA, David SG, Vaughan EE, Herzlinger DA (2001). Fibroblast growth factor-7 regulates stratification of the bladder urothelium. J Urol.

[24] Narla ST, Bushnell DS, Schaefer CM, Nouraie M, Bates CM (2020). Keratinocyte growth factor reduces Injury and leads to early recovery from Cyclophosphamide bladder Injury. Am J Pathol.

[25] Xu Q, Lin S, Wang Y, Zhu J, Lin Y (2018). Fibroblast growth factor 10 (FGF10) promotes the adipogenesis of intramuscular preadipocytes in goat. Mol Biol Rep.

[26] Finch PW, Cunha GR, Rubin JS, Wong J, Ron D (1995). Pattern of keratinocyte growth factor and keratinocyte growth factor receptor expression during mouse fetal development suggests a role in mediating morphogenetic mesenchymal-epithelial interactions. Dev Dyn.

