# Supplementary figures and images for: Distinct effects of Fgf7 and Fgf10 on the terminal differentiation of murine bladder urothelium revealed using an organoid culture system
Source: BMC Urol. 2023 Oct 24;23:169. doi: 10.1186/s12894-023-01338-y (PMC10594814; doi:10.1186/s12894-023-01338-y)

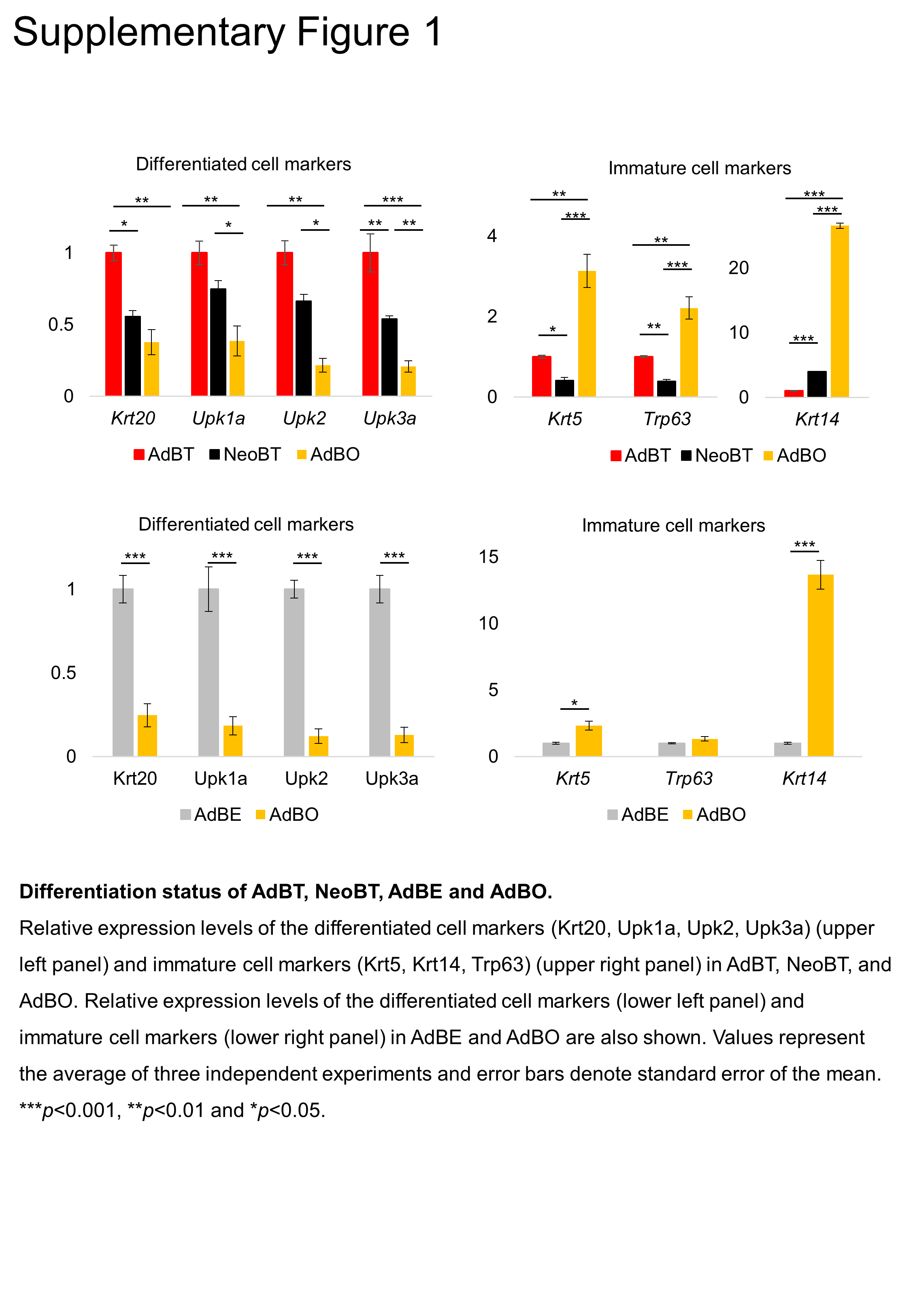

Supplement: Supplementary file 1 — Supplementary Material 1 [file 12894_2023_1338_MOESM1_ESM.png]

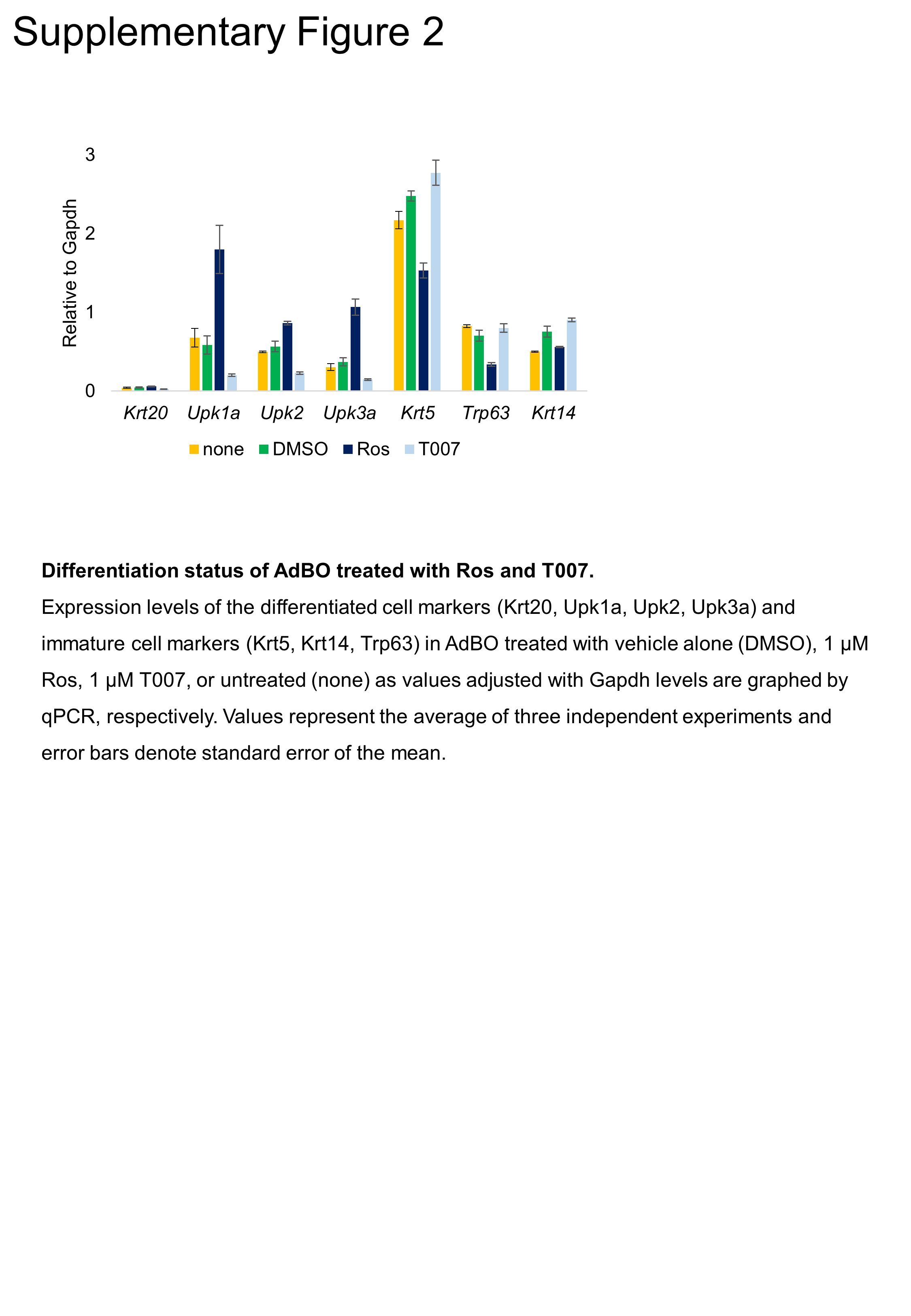

Supplement: Supplementary file 2 — Supplementary Material 2 [file 12894_2023_1338_MOESM2_ESM.png]
